# Use of Network Pharmacology to Investigate the Mechanism by Which Allicin Ameliorates Lipid Metabolism Disorder in HepG2 Cells

**DOI:** 10.1155/2021/3956504

**Published:** 2021-01-12

**Authors:** Bijun Cheng, Tianjiao Li, Fenglin Li

**Affiliations:** Jilin Agricultural Science and Technology University, Jilin, China

## Abstract

Allicin has been well documented to exhibit a wide spectrum of biological activities, especially lipid-lowering activity, as a promising candidate for the management of nonalcoholic fatty liver disease (NALFD). However, the mechanisms underlying the therapeutic effects of allicin require further investigation. It is tempting to think of combining network pharmacology and experimental validation to investigate the mechanism by which allicin ameliorates lipid metabolism disorder in HepG2 cells. We established a cell model of hepatic steatosis induced by PA to investigate the antisteatotic effects of allicin. The studies showed that allicin reduced PA-induced lipid accumulation using Nile red staining and TC and TG assays. Then, 219 potential targets of allicin were successfully predicted by PharmMapper. According to Reactome Pathway Analysis, 44 potential targets related to lipid metabolism were screened out. Molecular signaling cascades mediated by allicin included PPARA, PPARG, FABP4, and FABP6 by cytoHubba and qPCR analysis. Results revealed that allicin activated the gene expression of PPARA and FABP6 and suppressed the gene expression of FABP4 and PPARG. Thus, the present study united the methods of network pharmacology and experimental validation to investigate the protein targets of allicin on PA-induced lipid metabolism disorders to supply a reference for related application for the first time.

## 1. Introduction

Lipid metabolism disorders are common pathological processes in various clinical diseases and are characterized by abnormal changes in the content and/or arrangement of various kinds of lipoprotein [1], including elevations in total cholesterol (TC) and triglycerides (TG) [2]. Substantial evidence has demonstrated the association between lipid accumulation and systemic conditions, such as NALFD [3], hyperlipaemia [2], and type 2 diabetes mellitus [4]. Adjusting abnormal lipid metabolism is a practical procedure to slow or prevent the degeneration of systemic conditions. Simultaneously, it is recommended that advantageous dietetic components make an important impact on the prevention and therapy of lipid metabolism disorder [5].

Natural products have more and more widespread applications in medicine and green agriculture due to their mild and largely harmless properties in comparison with chemical agents. Allicin is produced by an enzymatic reaction when raw garlic is crushed or chopped. As an active constituent of garlic, allicin has been found to possess generous pharmacological activities, such as anticancer [6], antibacterial, antihypertensive, antihypolipidemic, and antihypoglycemic activities [7]. In addition, allicin also showed antioxidant role on Nile tilapia and stem cells. It has been proved in our previous research that allicin could reduce lipid droplets induced by 1,3-DCP in HepG2 cells, recommending allicin as a potential candidate for handling of abnormal lipid metabolism [8]. However, the mechanisms underlying the therapeutic effects of allicin require further investigation.

In this work, we aim to find the pharmacological mechanisms by which allicin ameliorates lipid metabolism disorder in HepG2 cells. First of all, we established a cell model of hepatic steatosis induced by PA to investigate the antisteatotic effects of allicin. Then, we predicted potential targets of allicin by PharmMapper, a free online tool applied to seek the target proteins for allicin on the basis of reverse pharmacophore mapping [8]. Furthermore, we conducted GO enrichment and pathway analysis for the potential targets of allicin. Moreover, protein–protein interaction (PPI) relationship between the target proteins was constructed by STRING. Then, the potential target proteins were further analyzed via qPCR. In the present study, we made an effort to elucidate the biological mechanisms by which allicin exhibits a lipid lowering activity using a combination of experimental operation and network pharmacology.

## 2. Materials and Methods

### 2.1. Reagents

Allicin and palmitic acid (PA) were purchased from Sigma Chemical Co. (St. Louis, MO, USA).

### 2.2. Cell Culture Experiments

HepG2 cells were purchased from ATCC. Cells were cultured under standard cell cultured conditions (DMEM, 10% FBS) at 37°C in a 5% CO_2_ atmosphere.

### 2.3. Cell Viability Assays

The cytotoxicity of PA and allicin was detected using CCK8 assays. 8 × 10^3^ cells per well were seeded in triplicate in 96-well plates overnight. First, cells were treated with various doses of PA and allicin for 22 h. Next, cells were treated with CCK-8 (Shangbao, China) for 2 h. The absorbance value of the culture solution in the plate was detected by a microplate reader (Bio Tek, USA) at 450 nm.

### 2.4. Determination of TC and TG Levels

TC and TG levels were analyzed by an enzymatic kit (Beckman Coulter, Inc.) and indicated as mM of TC and TG per milligram of cellular protein. The operations were performed following the kit manufacturer's instructions.

### 2.5. Nile Red Staining Assay

Briefly, 8 × 10^3^ cells per well were seeded in a 24-well plate overnight. Then, cells were treated with various doses of PA and allicin for 24 h. Nile red staining of cells was performed referring to the methods reported in the literature [9]. Cells were observed and pictured by a fluorescence microscope (BX53, Olympus, Japan).

### 2.6. Targets Predicted by PharmMapper

PharmMapper is a web server for potential drug target identification based on the use of a pharmacophore mapping approach. We obtained the sdf structure format of allicin from the PubChem database (CID: 65036). The molecular file of allicin was uploaded to the PharmMapper server. The search started using the maximum generated conformations at 300 by selecting “Human Protein Targets Only (v2010, 2241)” option and default value of 300 for the number of reserved matched targets as described previously [9, 10]. “Default Mode” was chosen for other parameters.

### 2.7. Functional Enrichment Analysis

DAVID provides a comprehensive set of functional annotation tools for investigators to understand the biological meaning behind a large list of genes. FunRich is a software mainly used for gene functional classification that provides a comprehensive set of functional annotation for researchers to understand biological characteristics [11]. In the present study, gene ontology (GO) function and Kyoto Encyclopedia of Genes and Genomes (KEGG) pathway enrichment analyses of allicin were performed through FunRich (version 3.1.3 for Windows) and DAVID databases (version 6.8).

### 2.8. PPI Network Construction

The Search Tool for the Retrieval of Interacting Genes (STRING; http://string.embl.de/) is a biological database designed to construct a PPI network of allicin based on the known and predicted PPIs and then analyze the functional interactions between proteins. Based on the STRING online tool in Cytoscape software (version 3.7.1), we obtained the PPI network data of proteins with a confidence score ≥ 0.7. Subsequently, the plug-in of cytoHubba in Cytoscape software was applied to explore the significant proteins in PPI network. The default settings were used for advanced options.

### 2.9. mRNA Analysis

Briefly, 5 × 10^5^ cells per well were seeded in a 6-well plate overnight. Then, cells were treated with various doses of PA and allicin for 24 h. RNA from cells was isolated using TRIZOL reagent (Invitrogen, Carlsbad CA, USA). RNA concentration was determined by the absorbance at 260/280 nm. cDNA was synthesized from 2 *μ*g of total RNA by 5 × All-In-OneMasterMix (abm, Canada). The primer sequences were designed using Oligo7 software (Table 1) and synthesized by Sangon Biotech Co. (Shanghai, China). The qRT-PCR reactions were set up by EvaGreen 2X qPCR MasterMix-No Dye (abm, Canada) according to the manufacturer's instructions. qPCR was performed by Bio-Rad CFX96 Touch Real-Time PCR detection system with the following cycle: 95°C for 10 min, followed by 95°C for 15 sec and 60°C for 60 sec for 40 cycles. At last, the dissolution curve was determined. The relative gene expression value was statistically evaluated by the 2^−ΔΔCt^ method.

### 2.10. Statistical Analysis

Statistical significance of difference in measured variables was performed by SPSS 19.0 software (SPSS Inc., Chicago, IL, USA). All data were analyzed by one-way ANOVA. Data are expressed as mean ± SEM and plotted in histograms with GraphPad Prism 5.0.

## 3. Results

### 3.1. Allicin Inhibits PA-Induced Hepatocyte Injury

**Figure 1:**
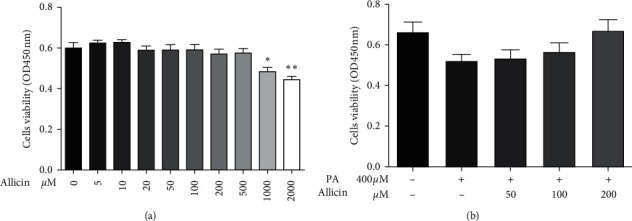
The effects of allicin and PA on cell viability in HepG2 cells. Cell viability was determined by CCK8 assays. The results were expressed as means ± S.D. of three independent experiments. ^*∗*^*p* < 0.05, ^*∗∗*^*p* < 0.01 vs. control; ^#^*p* < 0.05 vs. PA-treated cells.

First, we determined the concentration dependence of the cytotoxic effect of allicin in the absence or presence of 400 *μ*M PA for 24 h in HepG2 cells by using CCK8 assay. As shown in Figure 1(a), allicin (0–500 *μ*M) had no cytotoxic effect on HepG2 cells. As shown in Figure 1(b), allicin (50–200 *μ*M) prevented PA-induced cell death in HepG2 cells. Based on these results, concentrations from 50 *μ*M to 200 *μ*M were applied in the following studies.

### 3.2. Allicin Reduced Lipid Accumulation in PA-Induced Steatotic HepG2 Cells

To investigate the antisteatotic effect of allicin in HepG2 cells, HepG2 cells were exposed to various concentrations of allicin (50–200 *μ*M) in presence of a PA mixture at a concentration of 400 *μ*M for 24 h. Total intracellular lipid levels in HepG2 cells were measured after Nile red staining, and the TG and TC contents were assayed using enzymatic kits.

**Figure 2:**
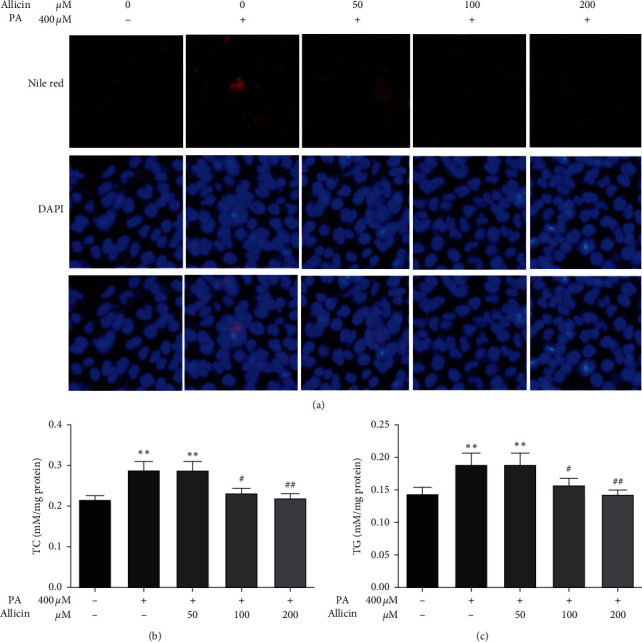
Inhibition of PA-induced lipid accumulation by allicin in HepG2 cells. (a) HepG2 cells were stained by Nile red and assessed by fluorescence microscopy (captured by microscope at 400X magnification). ((b) and (c)) TC and TG level were measured as described in the Materials and Methods section. The results are expressed as means ± S.D. of three independent experiments. ^*∗∗*^*p* < 0.01 vs. control; ^#^*p* < 0.05, ^##^*p* < 0.01 vs. PA-treated HepG2 cells.

From Figure 2(a), it is clear that cellular oil droplets were apparently increased by 400 *μ*M PA treatment and decreased in a concentration-dependent response of allicin treatment. In addition, the data of TC and TG validated the results of Nile red staining. A very significant reduction of TC and TG was observed in presence of allicin. The inhibitory effect of allicin on PA-induced lipogenesis of HepG2 cells was dose-dependent (Figures 2(b) and 2(c)).

### 3.3. Construction of the Interaction Network and Network Analysis

Ranked by fit score in descending order, 219 potential targets were predicted by PharmMapper. According to Reactome Pathway Analysis, 44 potential targets were found to be related to lipid metabolism. Subsequently, 44 potential targets were used for further investigation (Table 2).

### 3.4. GO Enrichment and Pathway Analysis for Potential Targets of Allicin

As a GO Term statistical analysis method, GO enrichment analysis focused on the biological function, molecular function, and cellular component [12, 13]. We imported the selected potential 44 target genes into the FunRich for GO enrichment. GO analysis results revealed that the functions of these potential targets were related to many biological processes that may be important for lipid metabolism, such as metabolism, energy pathways, transport, cell communication, and signal transduction. A total of 10 molecular functions were enriched, mainly involving transporter activity, catalytic activity, and ligand-dependent nuclear receptor activity. Cellular components related to lipid metabolism were also identified, including cytoplasm, nucleus, exosomes, extracellular region, and cytosol (Table 3).

**Figure 3:**
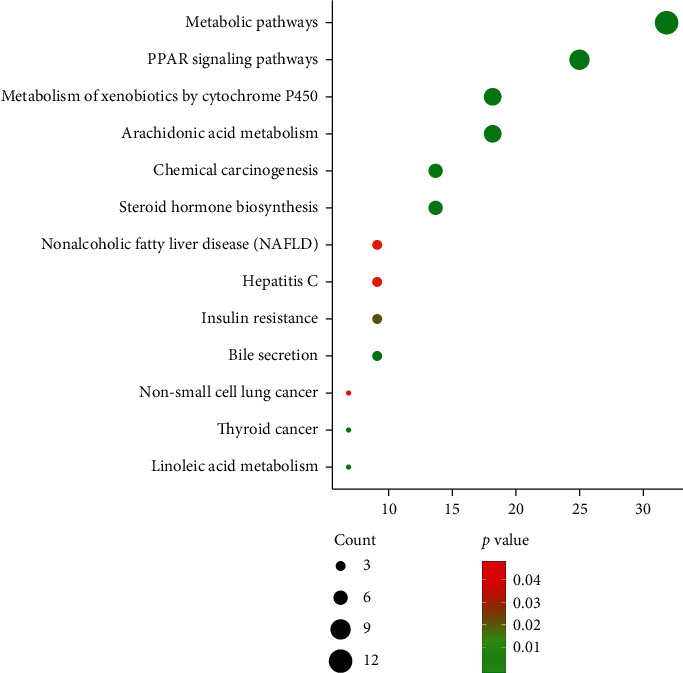
KEGG pathway enrichment of the potential targets.

Then, we imported the selected potential 44 target genes into the DAVID for pathway analysis. A total of 16 pathways were obtained by KEGG analysis, from which we acquired the top thirteen pathways that met the criterion of *p* < 0.05. These pathways contained PPAR signaling pathway, arachidonic acid metabolism, metabolism of xenobiotics by cytochrome P450, steroid hormone biosynthesis, chemical carcinogenesis, bile secretion, metabolic pathways, linoleic acid metabolism, thyroid cancer, insulin resistance, hepatitis C, non-small cell lung cancer, and nonalcoholic fatty liver disease (NAFLD). Among them, the most important enriched KEGG pathways were metabolic pathways and PPAR signaling pathway. The screened pathways suggested that allicin could play a role in the treatment with disorder of lipid metabolism by participating in the aforementioned pathways (Figure 3). In addition, genes involved in each pathway are listed in Table 4.

### 3.5. Protein-Protein Interaction Network (PPI) Analysis

**Figure 4:**
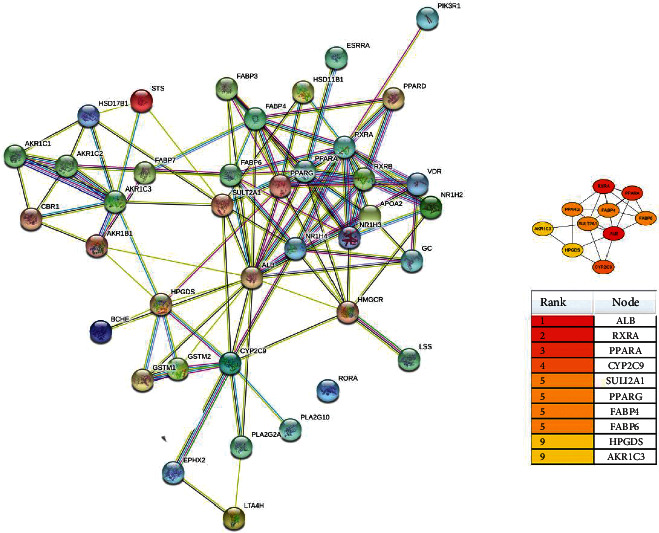
Candidate target proteins identified in the PPI network constructed using Cytoscape software.

**Figure 5:**
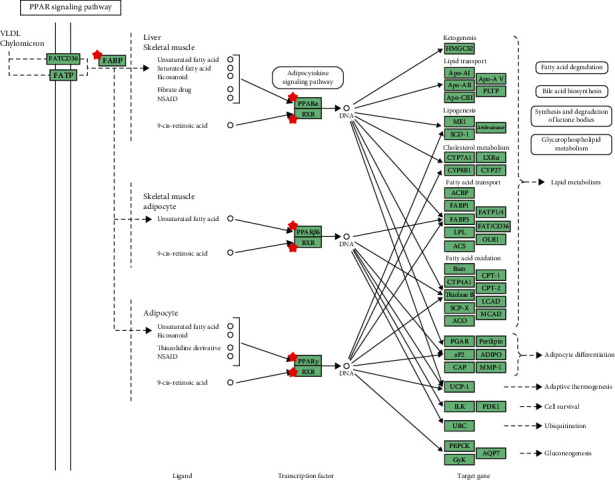
PPAR signaling pathway of potential targets of allicin.

The selected potential 44 target genes were imported into the STRING version 11.0 database in Cytoscape to construct a PPI network. The network complex included 40 nodes and 109 edges (Figure 4). Then, we applied cytoHubba in Cytotype to evaluate a node with a degree, which denotes the number of edges between a node and other nodes in a network. A high-degree node was the most influential node in the network, and a hub node was a component of a network with a high-degree node. In this study, we selected the top 10 hub nodes: ALB (degree = 19), RXRA (degree = 15), PPARA (degree = 11), CYP2C9 (degree = 10), SULT2A1 (degree = 9), PPARG (degree = 9), FABP4 (degree = 9), FABP6 (degree = 9), HPGDS (degree = 8), and AKR1C3 (degree = 8). The hub nodes maintain the stability of the network and show the metabolism of lipid, which involves multiple genes and multidimensional regulation. Among the hub nodes, RXRA, PPARA, PPARG, FABP4, and FABP6 belong to PPAR signaling pathway, which plays an important role in lipid metabolism (Figure 5). Therefore, RXRA, PPARA, PPARG, FABP4, and FABP6 were selected for subsequent analysis.

### 3.6. Validation of Potential Targets

**Figure 6:**
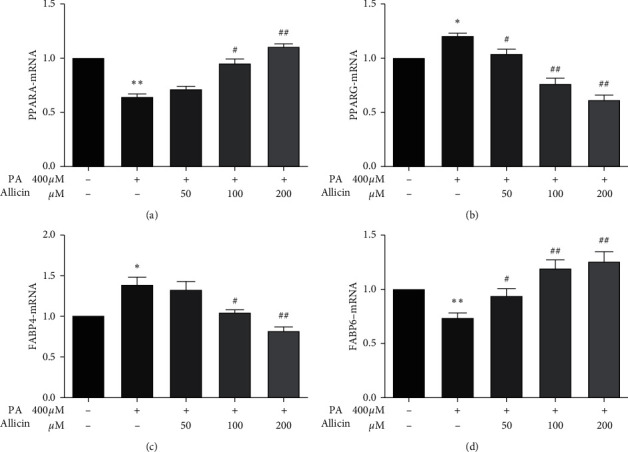
Effect of allicin on the relative gene expression in HepG2 cells. Values given are the mean ± SEM (*n* = 3). ^*∗∗*^*p* < 0.01, ^*∗∗*^*p* < 0.01 vs. control; ^#^*p* < 0.05, ^##^*p* < 0.01 vs. PA-treated HepG2 cells.

The effects of allicin on five candidate targets (RXRA, PPARA, PPARG, FABP4, and FABP6) were further investigated in HepG2 cells. We found no statistically significant difference in expression of RXRA mRNA. However, PPARA and FABP6 mRNA levels were significantly decreased by 400 *μ*M PA treatment (*p* < 0.01), while administration of allicin showed a concentration-dependent increase compared with PA group. Treatment with PA increased the expression of FABP4 and PPARG mRNA significantly, while administration of allicin lowered the expression of FABP4 and PPARG mRNA in a dose-dependent response (Figure 6).

## 4. Discussion

PharmMapper server is a freely accessed web server designed to identify potential target candidates for the given small molecules (drugs, natural products, or other newly discovered compounds with unidentified binding targets) using pharmacophore mapping approach [8, 9, 14]. The results of the network pharmacology method provide a basis for understanding the mechanism of action of allicin.

In the present study, we investigated the role of allicin in lipid metabolism. We found that allicin reduced lipid accumulation in a dose-dependent response in HepG2 cells. Then, using network pharmacology prediction, we successfully predicted 219 potential targets of allicin. According to Reactome Pathway Analysis, 44 potential targets related to lipid metabolism were screened out. Then, 44 potential targets were subjected to GO and KEGG pathway enrichment analyses.

GO analytical data suggested that the potential targets mainly referred to metabolism, energy pathways, transport, and cell communication. These biological processes were related to NAFLD and lipid metabolism, which were consistent with the literature [15, 16]. Therefore, the potential targets of allicin participated in multiple biological processes and played pivotal roles in lipid metabolism.

Based on the KEGG pathway analysis, potential targets were mainly involved in 16 pathways. Among them, PPAR signaling was an extremely important pathway. The PPAR signaling pathway has been considered to be related to the pathological processes of fatty liver development in rats with acute pancreatitis [17]. PPARA was a nuclear hormone receptor and important regulator of lipid metabolism in the liver. PPARA was considered to be an important regulator of lipid metabolism, which, upon activation, accelerated not only transport, binding, and *β*-oxidation of fatty acids, but also lipogenesis [18]. PPARA was mainly activated by ligand binding, which required heterodimer formation with RXR [19]. Another part of the PPAR family, PPARG, was a nuclear hormone receptor, which played an important role in the metabolism of lipids. In addition, it was also related to a variety of diseases, including diabetes, obesity, atherosclerosis, and cancer [20, 21]. It had been reported that PPARG could also be activated by fatty acids and played an important part in insulin sensitivity and fat production [22]. Therefore, in the present study, PPAR signaling pathway was supposed to serve crucial roles in the lipid metabolism regulated by allicin.

STRING database can analyze the interaction relationship between some proteins [23–25]. cytoHubba can identify hub objects and subnetworks from a complex interactome. Then, using of STRING database and cytoHubba suggested 10 hub targets, which play important roles in the PPI. Among them, RXRA, PPARA, PPARG, FABP4, and FABP6 belong to PPAR signaling pathway, which plays an important role in lipid metabolism. Therefore, RXRA, PPARA, PPARG, FABP4, and FABP6 included in PPAR signaling pathway were selected for further exploration by qPCR analysis. The present results revealed that allicin activated the gene expression of PPARA and FABP6 and suppressed the gene expression of FABP4 and PPARG. Findings are aligned with the previous studies. It was found that treatment with garlic essential oil (GEO) and diallyl disulfide (DADS) significantly upregulated the hepatic PPARA and CPT-1 expression levels in HFD-fed mice compared with HFD-fed mice without treatment [26]. A recent study also confirmed that PPARA activation enhances mitochondrial *β*-oxidation activity accelerating FA degradation in the liver [27]. A study performed confirmed the association between PPARG and obesity. It was reported that PPARG activation could normalize epigenetic and transcriptional regulation primarily related to lipid metabolism [28]. Moreover, PPARG induces the expression level of FABP4 leading to hepatic adipogenesis; the increase of fat is related to the upregulation of PPARG and FABP4 m RNA and downregulation of FABP6 mRNA [29–32]. Consistent with previous studies, our results indicate that allicin may alleviate the PA-induced lipid accumulation in HepG2 cells through activating the gene expression of PPARA and FABP6 and suppressing the gene expression of FABP4 and PPARG.

## 5. Conclusions

In conclusion, the underlying inhibitory mechanism of allicin on PA-induced lipogenesis of HepG2 cells was explored in this study by combining experimental operation and network pharmacology prediction. Moreover, the potential targets (PPARA, PPARG, FABP4, and FABP6) were successfully selected based on this practical strategy. Overall, our findings indicated that allicin might alleviate lipid accumulation in HepG2 cells, at least in part, through the PPAR signaling pathway.

## Figures and Tables

**Table 1:** Primer sequences used for qPCR.

Gene name	QRT-PCR primers	
RXRA	Forward	TCCTTCTCCCACCGCTCCATC
	Reverse	CAGCTCCGTCTTGTCCATCTG

PPARA	Forward	ATCCCATCACTCTCTCTGTG
	Reverse	AACTACCTGCTCAGGACTCA

PPARG	Forward	GCACTGCCTATGAGCACTTC
	Reverse	CCATTGGGTCAGCTCTTGTG

FABP4	Forward	TGGTGGTGGAATGCGTCAT
	Reverse	GGTCAACGTCCCTTGGCTTA

FABP6	Forward	AGCACCACCCATTCTCCTCA
	Reverse	AAGTGAAGTCCTGCCCATCCT

**Table 2:** Information of potential targets related to lipid metabolism.

Gene name			
FABP6	AKR1B1	AKR1C1	VDR
APOA2	LSS	NR1H2	HPGDS
ESRRA	PIK3R1	STS	HMGCR
FABP7	HSD17B1	CYP2C9	LTA4H
GM2A	ALB	PLA2G2A	DPEP1
FABP4	HSD11B1	MAPKAPK2	PCTP
FABP3	NR1H4	RXRB	PPARG
PPARA	NR1H3	BCHE	RORA
PPARD	AKR1C2	RXRA	PLA2G10
CBR1	AKR1C3	GC	PNMT
SULT2A1	EPHX2	GSTM1	GSTM2

**Table 3:** GO enrichment analysis of the potential targets.

	Term	Genes
Biological process	Metabolism	GM2A; CBR1; SULT2A1; AKR1B1; LSS; HSD17B1; HSD11B1; NR1H4; AKR1C2; AKR1C3; EPHX2; AKR1C1; STS; CYP2C9; PLA2G2A; BCHE; GSTM1; GSTM2; HPGDS; HMGCR; PLA2G10; PNMT
	Energy pathways	GM2A; CBR1; SULT2A1; AKR1B1; LSS; HSD17B1; AKR1C3; EPHX2; AKR1C1; STS; CYP2C9; PLA2G2A; BCHE; GSTM1; GSTM2; HPGDS; HMGCR; PLA2G10; PNMT
	Bone remodeling	RORA
	Transport	FABP6; APOA2; FABP7; FABP3; ALB; AKR1C2; GC; PCTP
	Xenobiotic metabolism	GSTM1
	Transcription	PPARD
	Regulation of gene expression, epigenetic	VDR
	Cell communication	FABP4; PIK3R1; NR1H4; NR1H3; NR1H2; MAPKAPK2; RXRB; RXRA
	Signal transduction	FABP4; PIK3R1; NR1H4; NR1H3; NR1H2; MAPKAPK2; RXRB; RXRA
	Protein metabolism	LTA4H; DPEP1

Molecular function	Ligand-dependent nuclear receptor activity	PPARA; NR1H4; NR1H3; NR1H2; RXRB; RXRA
	Transporter activity	FABP6; APOA2; FABP7; GM2A; FABP3; ALB; AKR1C2; GC; PCTP
	Catalytic activity	LSS; HSD17B1; HSD11B1; AKR1C3; AKR1C1; STS; CYP2C9; HMGCR
	Glutathione transferase activity	GSTM1; GSTM2
	Phospholipase activity	PLA2G2A; PLA2G10
	Oxidoreductase activity	CBR1; AKR1B1; AKR1C2
	Hydrolase activity	EPHX2; BCHE; LTA4H
	Transcription factor activity	PPARD; VDR; PPARG
	Protein serine/threonine kinase activity	MAPKAPK2
	DNA binding	RORA

Cellular component	Extracellular region	APOA2; ALB; PLA2G2A; BCHE; GC; PLA2G10
	Cytoplasm	FABP6; APOA2; FABP7; FABP4; FABP3; PPARA; CBR1; SULT2A1; AKR1B1; PIK3R1; HSD17B1; ALB; AKR1C3; EPHX2; AKR1C1; NR1H2; PLA2G2A; MAPKAPK2; GC; GSTM1; GSTM2; VDR; HPGDS; LTA4H; PCTP; PPARG
	Cytosol	SULT2A1; AKR1B1; PIK3R1; AKR1C2; EPHX2; AKR1C1; MAPKAPK2; PCTP; PNMT
	Exosomes	APOA2; GM2A; FABP3; CBR1; AKR1B1; ALB; EPHX2; GSTM2; LTA4H; DPEP1; PPARG
	Extracellular	APOA2; GM2A; FABP3; AKR1B1; ALB; PLA2G2A; BCHE; GC; HMGCR; PLA2G10
	Endoplasmic reticulum	LSS; HSD11B1; STS; CYP2C9; PLA2G2A; HMGCR; DPEP1
	Lysosome	APOA2; GM2A; CBR1; AKR1B1; LSS; HSD17B1; ALB; STS
	Extracellular space	AKR1B1; ALB; PLA2G2A
	Nucleus	ESRRA; PPARA; PPARD; PIK3R1; ALB; NR1H4; NR1H3; AKR1C3; NR1H2; MAPKAPK2; RXRB; RXRA; VDR; LTA4H; PPARG; RORA
	Plasma membrane	PIK3R1; STS; GC

**Table 4:** Genes involved in each pathway.

Term	Count	Genes
Metabolic pathways	14	PLA2G10, PNMT, HSD17B1, HMGCR, CYP2C9, EPHX2, LSS, AKR1C3, CBR1, AKR1B1, HSD11B1, PLA2G2A, LTA4H, HPGDS
PPAR signaling pathway	11	PPARA, APOA2, PPARD, RXRB, RXRA, PPARG, FABP3, FABP4, FABP7, FABP6, NR1H3
Arachidonic acid metabolism	8	AKR1C3, CBR1, PLA2G10, CYP2C9, PLA2G2A, EPHX2, LTA4H, HPGDS
Metabolism of xenobiotics by cytochrome P450	8	GSTM1, GSTM2, AKR1C2, CBR1, SULT2A1, CYP2C9, HSD11B1, AKR1C1
Steroid hormone biosynthesis	6	AKR1C3, AKR1C2, STS, HSD17B1, HSD11B1, AKR1C1
Chemical carcinogenesis	6	GSTM1, GSTM2, CBR1, SULT2A1, CYP2C9, HSD11B1
Bile secretion	4	SULT2A1, HMGCR, RXRA, NR1H4
Insulin resistance	4	NR1H2, PPARA, PIK3R1, NR1H3
Hepatitis C	4	PPARA, RXRA, PIK3R1, NR1H3
Nonalcoholic fatty liver disease (NAFLD)	4	PPARA, RXRA, PIK3R1, NR1H3
Linoleic acid metabolism	3	PLA2G10, CYP2C9, PLA2G2A
Thyroid cancer	3	RXRB, RXRA, PPARG
Non-small cell lung cancer	3	RXRB, RXRA, PIK3R1

## Data Availability

The data used to support the findings of this study are available from the corresponding author upon request.

## Abbreviations

CCK-8: Cell counting kit-8
TC: Total cholesterol
TG: Triglycerides
NALFD: Nonalcoholic fatty liver disease
PPARA: Peroxisome proliferator-activated receptor alpha
PPARG: Peroxisome proliferator-activated receptor gamma
FABP4: Fatty acid-binding protein
FABP6: Gastrotropin
qPCR: Quantitative real-time PCR.
